# Literary Notes

**Published:** 1905-12

**Authors:** 


### LITERARY NOTES.

THE National Standard Dispensatory, published by Lea Brothers & Company, Philadelphia, has made its appearance. It is a new work, covering the new pharmacopeia and the field of unofficial drugs. It is a most important addition to the armament of physician and pharmacist and will easily find its way into the workshops of both professions.

THE *Journal of Physical Therapy* has made its bow to the audience in the professional literary arena. Its first number, September, 1905, looks well and contains material of considerable interest. It is edited by Dr. Gustavus M. Blech, Chicago, and is published by Frank S. Betz, Hammond, Ind.

THE *State Board Journal of America* is the title of a new publication located at Washington, D.C., which appeared in September, 1905. It announces that it is devoted to the mutual interests of boards, students, and colleges of medicine, dentistry, and pharmacy. The first number contains much of import to each of these constituencies, among which may be mentioned the address of President Beates, at the fourteenth annual meeting of the National Confederation of medical examining and liscensing boards.

THE addresses at the dinner given to John B. Chapin, M.D., LL.D., in celebration of the completion of half a century in hospitals for the insane, have been published in book form. It is a handsome octavo of sixty-seven pages, and includes a portrait of Dr. Chapin, a list of the subscribers, a number of illustrations, the list of toasts and the after-dinner speeches. Dr. Edward N. Brush was the master of the feast, and interspersed his remarks in presenting the speakers with wit and humor that made the occasion memorable for its entertaining character. The dinner was given at the Bellevue-Stratford, Phialdelphia, December 1, 1904.

D. APPLETON & COMPANY, medical publishers, New York, announced that they would publish in November a new book entitled, Differential Diagnosis and Treatment of Disease. The author is Augustus Caillé, a fellow of the New York Academy of Medicine; Member and ex-president of the American Pediatric Society; professor of diseases of children, New York Post-

Graduate Medical School and Hospital: visiting physician to the New York Post-Graduate and German Hospitals; consulting physician to Isabella Home and Hospital.

---

THE *Transvaal Medical Journal,* located at Johannesburg, and published under the auspices of the Transvaal Medical Society, was isued in August, 1905. It is a well edited monthly magazine containing, among other valuable material, an epitome of current medical literature abstracted from all sources and classified under appropriate heads. It is a double column quarto of sixty pages, reflecting credit in every way upon its administrative officers.

---

THE *Alkaloidal Clinic,* which suffered recent destruction by fire of its machinery plant, will change its name with the January issue to The American Journal of Clinical Medicine.

THE *Illinois Medical Journal,* which is the official organ of the Illinois State Medical Society, is to move from Springfield to Chicago, January 1, 1906. In association with this change an improvement in form and appearance as well as increase in circulation is announced.

---

W. B. SAUNDERS & COMPANY, Philadelphia and London, have just issued a catalogue of Medical and Surgical books that is not only a fine specimen of the printer's art, but will prove helpful to every physician who buys books. It is handsomely illustrated and contains much descriptive text as well as many press notices and individual commendations.

---

D. APPLETON & COMPANY, New York, have just issued a complete catalogue of their medical books. It is illustrated with half-tone engravings of authors, drawings and other forms of art work of subjects, and contains specimen pages from many of their publications. It is one of the handsomest and most complete catalogues we have seen and will be suplied to any of our readers upon request to the publishers, 436 Fifth Avenue, New York.

---

A Textbook of Clinical Diagnosis, by Laboratory Methods. For the use of Students, Practitioners, and Laboratory Workers. By L. Napoleon Boston, A.M., M.D., Associate in Medicine and Director of the Clinical Laboratories at the Medico-Chirurgical College, Philadelphia. Second edition, revised and enlarged. Octavo of 563 pages, with 330 illustrations, including 34 plates, many in colors. Philadelphia and London: W. B. Saunders & Co. 1905. (Cloth, $4.00 net.; sheep or half-morocco, $5.00 net.)

The *Medical and Surgical Monitor* and the *Central States Medical Magazine* havė amalgamated under the name of the *Central States Medical Monitor.* Dr. S. E. Earp, formerly editor of the Central States, will be editor and Dr. S. P. Scherer, formerly editor of the Monitor, will be associate editor.

---

The *State Board Journal of America* is the title of a new publication located at Washington D. C., which appeared in September, 1905. It announces that it is devoted to the mutual interests of boards, students, and colleges of medicine, dentistry and pharmacy. The first number contains much of interest to each of these constituencies, among which may be mentioned the address of President Beates, at the fourteenth annual meeting of the National Confederation of Medical Examining and Licensing boards.

